# Efficacy and safety of lanreotide autogel compared with lanreotide 40 mg prolonged release in Chinese patients with active acromegaly: results from a phase 3, prospective, randomized, and open-label study (LANTERN)

**DOI:** 10.1186/s12902-020-0524-7

**Published:** 2020-05-04

**Authors:** Zhenmei An, Ting Lei, Lian Duan, Pei Hu, Zhongping Gou, Lihui Zhang, Lucie Durand-Gasselin, Nan Wang, Yan Wang, Feng Gu, on behalf of the LANTERN study investigators, Z. M. An, Z. P. Gou, J. W. Li, H. W. Tan, Q. Peng, L. B. Liang, Y. J. Li, P. Feng, L. Zheng, T. Lei, H. Q. Zhang, C. X. Li, C. Gan, Y. Xu, F. Gu, P. Hu, L. Duan, X. Chen, J. Li, W. G. Zhu, L. H. Zhang, S. Y. Zhang, F. Zhang, J. Li, Y. B. Li, H. J. Wang, Z. G. Mao, Z. H. Liao, Z. Y. Chen, Y. Mao, S. Q. Li, Y. Zhao, M. Shen, Nanjing T. Yang, X. Q. Zheng, Z. X. Wang, R. Gu, Guiyang L. X. Shi, M. Zhang, B. H. Xiao, J. M. Hou, J. P. Wen, Y. Lin, X. H. Lan, W. Lin, J. X. Liang, M. Zhu, H. W. Jia, J. Cui, F. A. Li

**Affiliations:** 1 West China Hospital, Sichuan University, Chengdu, Sichuan China; 2 Tongji Hospital, Tongji Medical college of HUST, Wuhan, China; 3 Peking Union Medical College Hospital, PUMCH, No.1 Shuaifuyuan, Wangfujing, Dongcheng District, Beijing, 100730 China; 4 The second hospital of Hebei Medical University, Shijiazhuang, China; 5 Ipsen Innovation, Les Ulis, France; 6 Ipsen Pharma, Beijing, China

**Keywords:** Acromegaly, Lanreotide autogel, Lanreotide, Somatostatin analogs, Chinese patients, Clinical trial

## Abstract

**Background:**

Lanreotide autogel is a somatostatin analog (SSA) approved for the treatment of acromegaly in 73 countries worldwide; however, it is not yet approved in China. The aim of this study was to evaluate the efficacy and safety of lanreotide autogel compared with lanreotide 40 mg prolonged release (PR) in Chinese patients with active acromegaly.

**Methods:**

LANTERN was a phase 3, randomized, open-label, non-inferiority study. Patients with active acromegaly who had undergone surgery ≥3 months prior, or were unlikely or unable to undergo surgery, were treated with lanreotide autogel 60/90/120 mg (monthly deep subcutaneous injection) or lanreotide 40 mg PR (intramuscular injection every 7, 10, or 14 days) for 32 weeks. Primary endpoint was mean change-from-baseline in age-adjusted insulin-like growth factor-1 (IGF-1) standard deviation scores (SDS) at the end-of-study. Secondary endpoints included: growth hormone (GH) levels ≤2.5 μg/L or ≤ 1.0 μg/L, ≥20% reduction in tumor volume (TV) and safety.

**Results:**

In total, 128 patients were randomized and received study treatment. Lanreotide autogel was non-inferior to lanreotide 40 mg PR: treatment difference (95% CI) for IGF-1 SDS between groups was − 0.32 (− 0.74, 0.11; per protocol population) and − 0.27 (− 0.63, 0.09; intention-to-treat [ITT] population), respectively. Reductions in IGF-1 (− 6.453 vs − 7.003) and GH levels (− 9.548 μg/L vs − 13.182 μg/L), and the proportion of patients with ≥1 acromegaly symptom (− 20.3% vs − 32.5%) were observed from baseline to end-of-study in lanreotide autogel and lanreotide 40 mg PR groups, respectively. In the lanreotide autogel group, 45.5% (25/55) patients achieved ≥20% reduction in TV compared with 50.9% (25/53) in lanreotide 40 mg PR group (ITT). Safety profiles were similar in both treatment groups.

**Conclusions:**

Lanreotide autogel was non-inferior to lanreotide 40 mg PR in Chinese patients with active acromegaly after 32 weeks of treatment.

**Trial registration:**

Retrospectively registered on ClinicalTrials.gov: NCT02493517 (9 July 2015); prospectively registered on chinadrugtrials.org.cn: CTR20140698 (24 October 2014).

## Background

Treatment goals for acromegaly include normalization of biochemical variables (growth hormone [GH] and insulin-like growth factor-1 [IGF-1] levels), tumor volume reduction (when caused by a pituitary tumor), improvement of signs, symptoms and comorbidities of the disease, and minimization of long-term mortality risk [1]. For most patients, transsphenoidal surgery is the primary treatment route [2]. Although complete surgical resection is possible, only 27 to 74% of patients have been reported to achieve complete remission, and consequently are able to delay or reverse the progression of complications such as diabetes [3]. For patients who do not go into complete remission following surgery, or who are poor surgical candidates, medical therapy is an alternative option to counter excess GH and IGF-1 [2, 4].

Somatostatin analogs (SSAs) are the preferred type of medical therapy used to reduce GH secretion. Patients who have persistent disease following surgical resection are recommended treatment with the first-generation long acting SSAs, octreotide long-acting release (LAR) or lanreotide autogel [4]. Lanreotide is a synthetic octapeptide analog of natural somatostatin with a longer half-life (t_½_) than the native molecule, and a selective somatostatin receptor subtype agonist with a high affinity for human somatostatin receptors 2 and 5 [5], which are commonly expressed at both the mRNA [6] and protein [7] level in GH-secreting pituitary tumors. A microparticle formulation of lanreotide (40 mg intramuscular injection every 7, 10 or 14 days depending upon the patient’s response to treatment) was initially developed to extend the duration of release, followed by lanreotide autogel, which supports a 28-day (or longer) dosing interval [8–10]. The efficacy and safety of lanreotide autogel for the medical management of acromegaly has been demonstrated in various international studies [11–17]. The efficacy of lanreotide autogel has not only been demonstrated in terms of hormonal control and reduction of acromegaly symptoms [11, 13, 15–18], but also in tumor shrinkage [17].

Lanreotide autogel is approved for the treatment of acromegaly in 73 countries, but is not yet approved for this indication in China. The aim of this study was to evaluate the efficacy and safety of lanreotide autogel 60, 90 or 120 mg compared with lanreotide 40 mg prolonged release (PR) in Chinese patients with active acromegaly.

## Methods

### Patients

Male and female patients aged 18 to 75 years, with active acromegaly, defined as elevated IGF-1 (≥1.3 × upper limit of normal [ULN]) and GH levels (mean ≥ 2.5 μg/L; average calculated from samples at 0, 30, 60, 90, and 120 min during the screening period) were eligible to participate in the study. Patients were included if they had either surgical removal of the adenoma ≥3 months before screening, or were likely to require pituitary surgery at a later time, but not before completing ≥32 weeks of study treatment (plus an additional 8 weeks for patients in the pharmacokinetic [PK] subgroup), or if pituitary surgery was deemed not an option. For patients receiving replacement therapy with thyroid hormones, levels of thyroid hormones were required to be within the normal range for ≥1 month before screening. For patients receiving corticosteroids and/or sex hormones, the dose of these was required to be stable for ≥1 month before screening.

Patients were excluded if they had received treatment with radiotherapy within the previous 10 years, or if a need for radiotherapy during the study was anticipated. Also excluded were patients who received treatment with short-acting octreotide or bromocriptine within the preceding 2 weeks, lanreotide autogel, lanreotide (lanreotide 40 mg PR; lanreotide PR microparticle), pegvisomant, cabergoline or octreotide LAR within the preceding 3 months, or traditional Chinese medicine within the preceding month. Other reasons for exclusion included use of investigational medicinal products or devices within ≤90 days previously, and/or being scheduled to receive such treatment during the study, defects in visual field according to clinical judgement, or unsuitability for magnetic resonance imaging (MRI). Patients with major cardiovascular diseases, uncontrolled diabetes (glycated hemoglobin [HbA_1c_] > 8.5%), symptomatic gallbladder disease, current concomitant malignancies, or clinically significant renal (creatinine > 1.5 × ULN) or hepatic disease (any liver enzyme > 2.5 × ULN) were excluded. In addition, patients with any severe, acute, or chronic medical or psychiatric condition or any laboratory abnormality that might have increased the risk associated with study participation or lanreotide administration, and made the patient inappropriate for entry into the study in the judgement of the investigator, or have interfered with the interpretation of the study results, were excluded. Lactating women and those likely to become pregnant were excluded, as too were patients with hypersensitivity to the study medications.

The PK subgroup included patients randomized to lanreotide autogel who were attending either of two selected study sites (Peking Union Medical College Hospital [PUMCH], Beijing, China or West China Hospital, Sichuan University, Sichuan, China).

### Study design and interventions

**Fig. 1:**
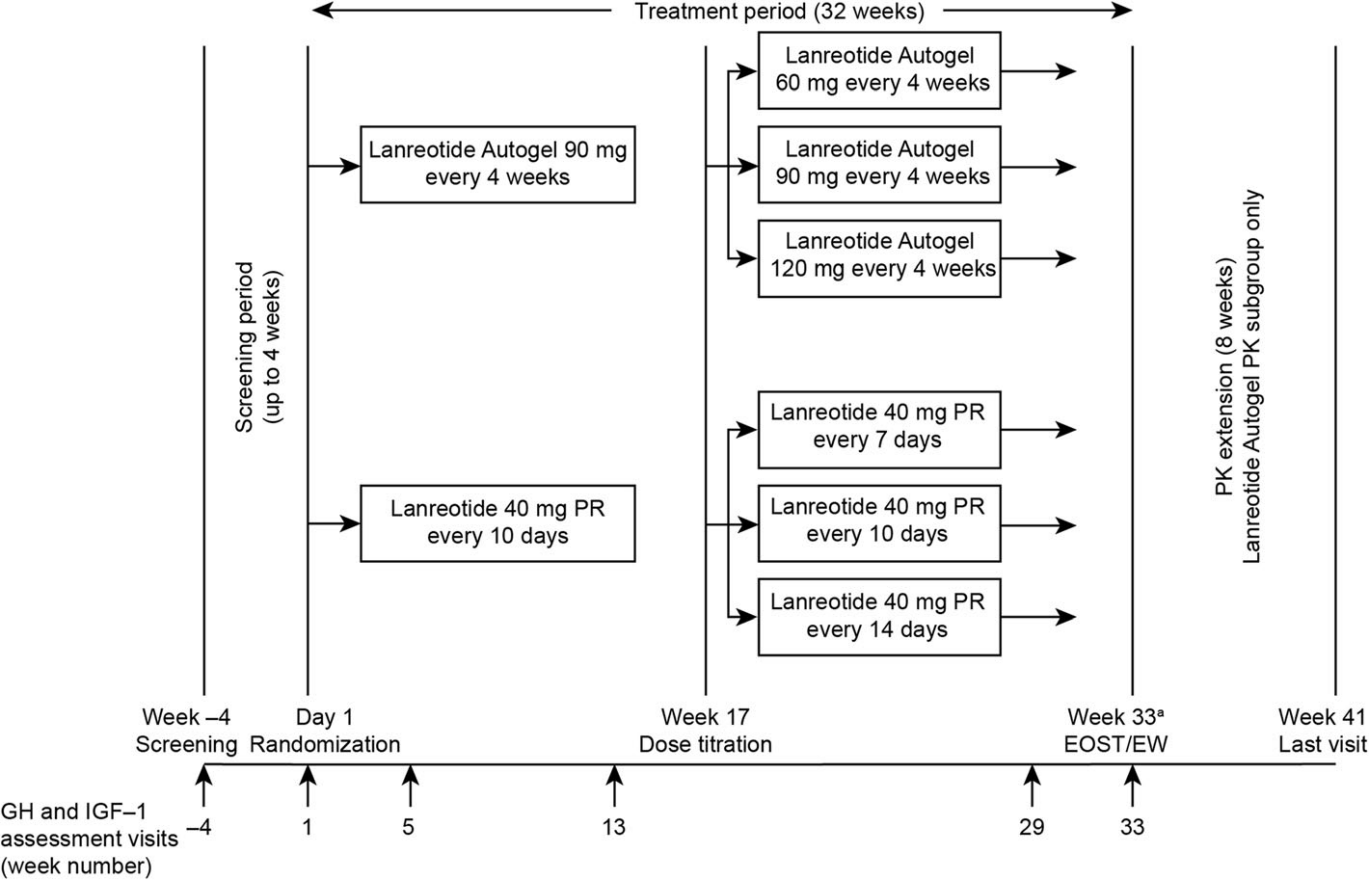
Study design. ^a^The planned EOST visit was on Week 33 (Day 225) for subjects in lanreotide autogel group and on Week 32 (Day 221 or Day 223 depending on dosing regimen) for subjects in lanreotide 40 mg PR group. EOST/EW, End of Study/ Early Withdrawal Visit; GH, growth hormone; IGF-1, insulin-like growth factor-1; PK, pharmacokinetics; PR, prolonged release

LANTERN was a phase 3, prospective, randomized, stratified, parallel-group, non-inferiority study comparing the efficacy and safety of lanreotide autogel 60, 90, or 120 mg with lanreotide 40 mg PR (ClinicalTrials.gov: NCT02493517; chinadrugtrials.org.cn: CTR20140698; Fig. 1). The study was conducted between 29 October 2014 and 16 February 2017. As the treatments differed in method and frequency of administration, the study was conducted on an open-label basis. There were 32 weeks of study treatment (all patients) and an 8-week extension phase without study treatment for further assessment of PK in a subgroup of patients receiving lanreotide autogel. The study was conducted in 10 specialist centres in China, including eight endocrinology sites and two neurosurgery sites. Patients were enrolled by investigators and randomized, using a computer-generated list (prepared by a sponsor-independent statistician) to lanreotide autogel or lanreotide 40 mg PR in a 1:1 ratio, with stratification based on the presence or absence of previous pituitary surgery. Investigators obtained randomization codes through an interactive web response system.

Patients received lanreotide autogel 90 mg every 4 weeks (deep subcutaneous injection) up to Week 13, or lanreotide 40 mg PR every 10 days (intramuscular injection) up to Week 15. IGF-1 and mean GH levels were measured at Week 13 to assess each patient’s response and guide dose titration (from Week 17 for those receiving lanreotide autogel and Week 16 for those receiving lanreotide 40 mg PR; Fig. 1). For up-titration of lanreotide autogel to 120 mg every 4 weeks or lanreotide 40 mg PR every 7 days, GH levels had to be > 2.5 μg/L or IGF-1 levels >ULN; for down-titration of lanreotide autogel to 60 mg every 4 weeks or lanreotide 40 mg PR every 14 days, GH levels had to be ≤1.0 μg/L and IGF-1 levels normalized.

Treatment then remained unchanged until the end of the study. Patients in the PK subgroup discontinued lanreotide autogel during the PK extension phase, but could receive octreotide as rescue medication, if required.

### Study visits and assessments

Study visits and assessments are detailed in Fig. 1.

#### Hormone and tumor assessments

IGF-1 samples were assayed using Chemiluminescence (Siemens Healthcare GmbH, Germany) at screening, weeks 1, 5, 13, and at either week 28 (patients receiving lanreotide 40 mg PR every 14 days) or week 29 (lanreotide autogel, lanreotide 40 mg PR every 10 days and lanreotide 40 mg PR every 7 days), and at the end-of-study/early withdrawal visit (pre-dose under fasting conditions except week 1, which was taken after study treatment). GH levels were assayed using electrochemiluminescence (ECL) and the automated MODULAR ANALYTICS E 170 (both Roche, Basel, Switzerland) using standard calibration against recombinant GH (international reference preparation 98/574). Fasting mean GH levels, calculated from a 2-h fasting GH cycle (five consecutive samples taken at 0, 30, 60, 90, and 120 min) were assessed at screening, week 13, week 29 (patients receiving lanreotide autogel only) and at the end-of-study/early withdrawal visit (pre-dose in all cases). Random GH levels were assessed at weeks 1 and 5, then at weeks 28 or 29 (as for IGF-1 levels) (pre-dose, except week 1). IGF-1 and GH levels were determined centrally from blood samples.

Tumor volumes (solid component) were measured centrally by two independent readers (using prespecified methods; Bioclinica, Newark, California, and Ipsen Pharma, Les Ulis, France) from MRI scans obtained at screening and at the end-of-study/early withdrawal visit. Readers were blind to sequence of scans and patients’ treatment allocation. The readers measured tumor volumes by drawing tumor contours on each MRI image where the adenoma was visible; the volume was then automatically calculated by Smart Study Direct (version 9) software (Bioclinica, Newark, California), taking contour areas and slice thickness into account. Solid and liquid (cyst-like) components of the adenoma were quantified separately. In case of discrepancies between readers, a third reader adjudicated. Acromegaly symptoms (headache, excessive perspiration, fatigue, soft-tissue swelling, and arthralgia) were graded (absent, mild, moderate, severe) at screening, Week 13 and at the end-of-study/early withdrawal visit.

#### Glycaemia and HbA_1c_ assessments

All clinical laboratory tests were performed by a central laboratory (LabCorp, Burlington, North Carolina). HbA_1c_ was measured using high-performance liquid chromatography by Bio-Rad Variant II Turbo Hemoglobin assay system to measure HbA_1c_.

#### PK assessments

Lanreotide autogel – pre-dose serum samples were taken at weeks 1, 13, 17, and 29; sampling around 4 h after treatment at weeks 13 and 17 and at the end of study (Week 33)/early withdrawal visit. For a subset of patients in the PK subgroup, additional samples were taken during the study and during the PK extension phase: after the first administration of 90 mg at Week 1 (1, 4, 6, 8, 12, 24, and 72 h), Week 2 (168 h), Week 3 (336 h), Week 4 (504 h) and prior to the next administration at Week 5 (672 h); after the fourth (last) administration of the titrated dose at Week 29 (1, 4, 6, 8, 12, 24, and 72 h), Week 30 (168 h), Week 31 (336 h), Week 32 (504 h), and at Week 37 (1344 h) and Week 41 (2016 h).

Lanreotide 40 mg PR – pre-dose serum samples were taken at weeks 1, 2, 13, 16, and at the end-of-study/early withdrawal visit (all taken shortly before treatment) for all patients.

#### Safety parameters

Treatment-emergent adverse events (TEAEs) were monitored throughout the study. Vital signs were assessed, and physical examinations and clinical laboratory tests performed at screening, Week 13 and at the end-of-study/early withdrawal visit. Gallbladder echography and electrocardiograms (ECG) were performed at screening, Week 13 (ECG only) and at the end-of-study/early withdrawal visit. Any clinically significant findings on the ECG, in the opinion of the investigator, were recorded as adverse events (AEs).

As recommended by regulatory guidelines, a multi-tiered approach was used to test for the presence of anti-drug (lanreotide) antibodies (ADA). Sera samples were assessed centrally at weeks 1 and 13, and at the end-of-study/early withdrawal visit using an electrochemiluminescence assay for the detection of binding ADAs. This assay used a bridging assay format, whereby labelled forms (with biotin and SulfoTAG) of lanreotide were used as tracers. Briefly, sera samples were incubated with biotin-lanreotide and SulfoTAG-lanreotide; after the incubation period, where binding equilibrium was reached, the mixture was transferred to a streptavidin-coated Meso Scale Discovery plate. Immune complexes composed of biotin-lanreotide, anti-lanreotide antibodies and SulfoTAG-lanreotide were captured by the streptavidin plate and measured by electrochemiluminescence. Positive samples identified in the screening electrochemiluminescence assay were submitted to a confirmatory electrochemiluminescence assay. These ADA positive samples were further analyzed using a titration assay to determine the titer value (reciprocal dilution at the highest dilution greater or equal to the screening cut point).

### Endpoints

The primary endpoint was the mean change-from-baseline in age-adjusted IGF-1 level (expressed as standard deviation score [SDS]) at the end-of-study/early withdrawal visit. Secondary efficacy endpoints included proportions of patients achieving normalized IGF-1 levels, GH levels ≤2.5 μg/L, GH levels ≤1.0 μg/L, and normalized IGF-1 levels with 1.0 μg/L < GH levels ≤2.5 μg/L; changes in GH levels; proportions of patients with ≥20% reduction in tumor volume; changes in tumor volume; proportions of patients with at least one symptom of acromegaly compared with baseline; and safety. Analyses of efficacy endpoints by treatment regimen (ie, lanreotide autogel 60, 90 and 120 mg or lanreotide 40 mg PR 7, 10 and 14 days) was a defined secondary efficacy endpoint. However, as a result of the small numbers of patients receiving some regimens, it was not possible to interpret findings and these data are not considered within this manuscript.

PK endpoints included trough lanreotide concentrations (C_trough_ i.e. observed serum concentration at the end of the dosing interval) prior to the fourth, fifth and eighth administrations and at the end-of-study treatment visit with lanreotide autogel, and prior to the second, tenth and twelfth administrations and at the end-of-study visit with lanreotide 40 mg PR. Following a similar approach as reported by Bronstein and colleagues, a non-compartmental PK analysis was performed for the lanreotide autogel PK subgroup to provide individual PK parameters over a 4-week dosing interval after the first and last administration of study drug (i.e. steady state): maximum plasma concentration (C_max_), time to maximum plasma concentration (t_max_), area under the concentration–time curve over a 4-week dosing interval (AUC_tau_), C_trough_ and elimination t_½_ [19].

Additional parameters were estimated, comparing lanreotide PK after Week 1 and Week 29 after normalization to a dose of 90 mg: accumulation ratio for C_trough_ (R_trough_) = C_trough_,Week 29/C_trough_,Week 1; accumulation ratio for C_max_ (R_max_) = C_max_,Week 29/C_max_,Week 1; accumulation ratio for AUC_tau_ (R_AUC_) = AUC_tau_, Week 29/AUC_tau_, Week 1.

### Statistical analyses

Statistical analyses were performed using SAS® version 9.3 (SAS Institute Inc., Cary, NC, USA). Non-inferiority was declared if the upper limit of the two-sided 95% confidence intervals (CI) for the treatment difference in the primary endpoint (lanreotide autogel – lanreotide 40 mg PR) was less than the non-inferiority margin (i.e. 0.6 log [SDS] for least squares [LS] mean difference of log [base e] IGF-1 SDS values between treatment groups resulting from the generalized linear model [GLM]), and 50 patients per group were required for 81% power. Assuming that approximately 85% of the patients randomized were evaluated in the per protocol (PP) population, 59 patients per group were required to be randomized. Therefore to ensure at least 100 patients in the PP population, a total of 118 patients were needed for randomization (intention-to-treat [ITT] population).

The ITT population included all randomized and treated patients having ≥1 baseline and ≥ 1 post-baseline assessment of the primary efficacy parameter. The PP population included all ITT patients for whom no major protocol deviations occurred with impact on efficacy assessment. The safety population comprised all randomized patients who received at least one dose of study drug. The primary efficacy endpoint was the mean change-from-baseline in age-adjusted IGF-1 values (expressed as log-transformed SDS values) at the end-of-study/early withdrawal visit in patients treated with lanreotide autogel and lanreotide 40 mg PR. For each patient, the respective IGF-1 SDS value was calculated based on the z-score derivation: IGF-1 SDS = (IGF-1 – mean) / (standard deviation; SD), with mean and SD derived from the ULN and lower limit of normal (LLN) margins provided by the central laboratory for each age category (LLN = mean – 2 SD; ULN = mean + 2 SD). Secondary efficacy endpoints were analyzed using descriptive statistics and tabulated by treatment group for the ITT population only. For safety data, baseline values were defined as the last measurement of the specific safety variables collected before the first dose of study treatment. In general, descriptive statistics were used. All efficacy endpoints were analyzed at end-of-study/early withdrawal visit, corresponding to a last observation carried forward imputation approach for early withdrawal. No other imputations were performed for missing data. For safety endpoints, if a value required a retest (for clinical laboratory, vital signs, ECGs, gallbladder echography), the closest non-missing reliable value to the scheduled visit was used in the summary tables. A PK/pharmacodynamic analysis for lanreotide autogel, describing the relationship between serum lanreotide levels and GH and IGF-1 compared with GH levels, was performed where possible using NONMEM software (version 7.3).

This manuscript adhered with CONSORT guidelines/methodology.

## Results

### Patient disposition

**Fig. 2:**
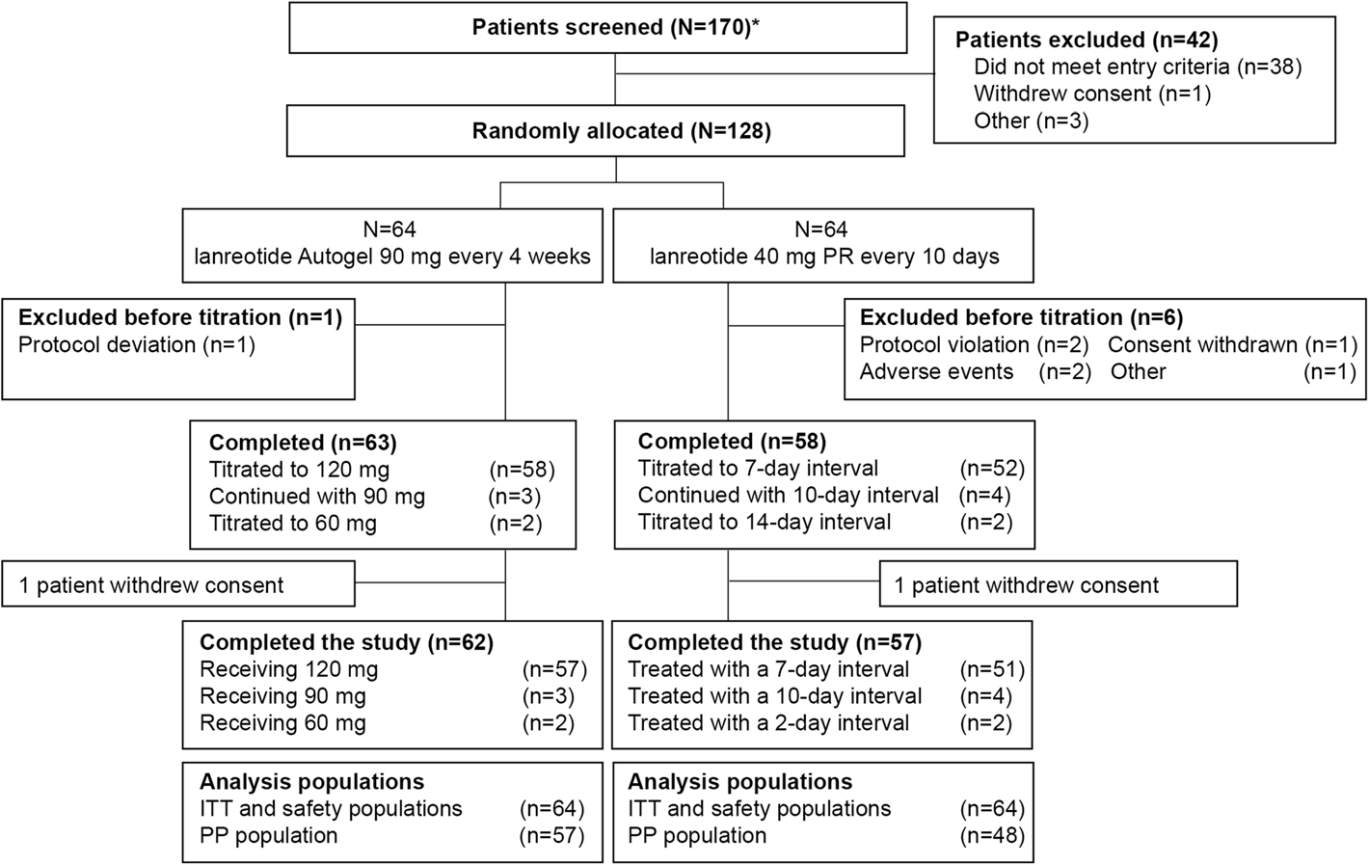
Patient disposition. PP, per-protocol; PR, prolonged release; ITT, intention-to-treat. *Includes re-screened patients (*n* = 15)

In total, 170 patients were screened and 128 were randomly allocated to receive treatment (lanreotide autogel, *n* = 64; lanreotide 40 mg PR, *n* = 64) (Fig. 2) and included in the ITT and safety populations. Following the fixed dose stage, patients received titrated doses of lanreotide autogel: 58 patients were up-titrated to 120 mg, three patients continued to receive 90 mg, two patients were down-titrated to 60 mg and one patient was excluded before titration. In the lanreotide 40 mg PR group, 52 patients were up-titrated to every 7 days, four patients continued to receive lanreotide 40 mg PR every 10 days, two patients were down-titrated to every 14 days and six patients were excluded before titration. Overall, 23 patients were excluded from the PP population: seven in the lanreotide autogel group (major procedure deviations [*n* = 5]; major protocol deviations of Good Clinical Practice [GCP] breach [*n* = 2]) and 16 in the lanreotide 40 mg PR group (procedure deviations [*n* = 12]; major protocol deviations of GCP breach [*n* = 2]; incorrect study treatment [*n* = 1]; prohibited concomitant medication [*n* = 1]). In total, 119 (93.0%) patients completed the study (lanreotide autogel, *n* = 62; lanreotide 40 mg PR, *n* = 57).

### Patient demographics and baseline characteristics

Baseline demographic and disease characteristics were similar for both treatment groups and are summarized in Table 1. The majority of patients were female in both treatment groups, and most had macroadenomas (lanreotide autogel, *n* = 43 [67.2%]; lanreotide 40 mg PR, *n* = 47 [73.4%]) and had undergone pituitary surgery previously (both groups, *n* = 44 [68.8%]). The most frequently reported medical histories were hypertension (both groups, *n* = 13 [20.3%]), sinusitis (lanreotide autogel *n* = 8 [12.5%]; lanreotide 40 mg PR, *n* = 10 [15.6%]), diabetes mellitus (lanreotide autogel, *n* = 9 [14.1%]; lanreotide 40 mg PR, *n* = 7 [10.9%]), hyperlipidemia (lanreotide autogel, *n* = 8 [12.5%]; lanreotide 40 mg PR, *n* = 6 [9.4%]) and thyroid mass nodule (lanreotide autogel, *n* = 4 [6.3%]; lanreotide 40 mg PR, *n* = 8 [12.5%]).

**Table 1:** Baseline demographic and disease characteristics

	**Lanreotide autogel** (***N*** **= 64**)	**Lanreotide 40 mg PR** (***N*** **= 64**)	**All patients** (***N*** **= 128**)
**Age, years**			
Mean (SD)	37.9 (11.2)	40.8 (12.6)	39.4 (11.9)
**Sex, n (%)**			
Male	26 (40.6)	24 (37.5)	50 (39.1)
Female	38 (59.4)	40 (62.5)	78 (60.9)
**BMI, kg/m**^**2**^			
Mean (SD)	26.3 (3.6)	26.1 (3.6)	26.2 (3.6)
**Time since acromegaly, years**			
Mean (SD)	3.70 (5.83)	3.14 (4.09)	3.42 (5.02)
**Previous treatment, n (%)**			
Pituitary surgery	44 (68.8)	44 (68.8)	88 (68.8)
Radiotherapy	3 (4.7)	3 (4.7)	6 (4.7)
**Any acromegaly medication ≤ 6 months prior to study entry, n (%)**	10 (15.6)	12 (18.8)	22 (17.2)
**Tumor assessment at baseline by central evaluation, n (%)**	(*n* = 63)	(*n* = 64)	(*n* = 127)
Microadenoma^a^	15 (23.4)	12 (18.8)	27 (21.1)
Macroadenoma^a^	43 (67.2)	47 (73.4)	90 (70.3)
No tumor	5 (7.8)	5 (7.8)	10 (7.8)^b^
**Solid component (μL)**	(*n* = 58)	(*n* = 57)	(*n* = 115)
Mean (SD)	4990.5 (8752.8)	2589.1 (2666.5)	3800.2 (6576.7)

Data are presented from the intention-to-treat population. ^a^Tumor diameters: ≤10 mm were classified as microadenomas and > 10 mm as macroadenomas. ^b^Patients who had no tumors at baseline had GH levels ranging from 3.3–24.3 μg/L in the lanreotide autogel group (*n* = 5), and 2.8–13.0 μg/L in the lanreotide 40 mg PR group (*n* = 5). *BMI* body mass index, *GH* growth hormone, *PR* prolonged release, *SD* standard deviation

### Efficacy

#### Log-transformed age-adjusted IGF-1 SDS values

**Fig. 3:**
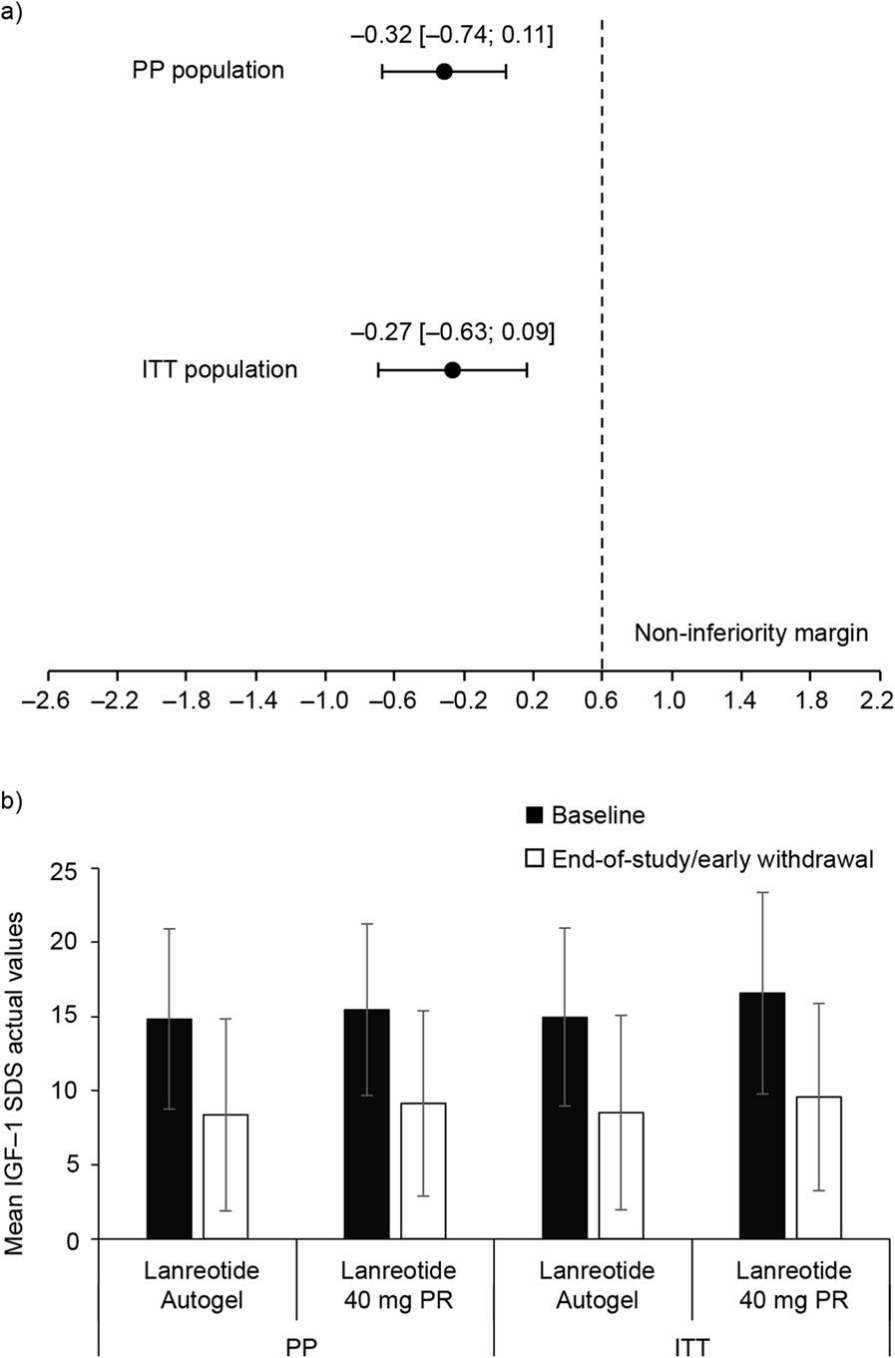
IGF-1 SDS values for lanreotide autogel and lanreotide 40 mg PR. a) Treatment differences for LS mean changes between baseline and end-of-study/early withdrawal visit (primary endpoint). b) Age-adjusted actual values during the study. Data in figure a are LS mean treatment differences (lanreotide autogel minus lanreotide 40 mg PR) [95% CI] from age-adjusted log-transformed values and based on a generalized linear model with IGF-1 SDS change as dependent variable, treatment group and previous surgery as independent factors, and baseline IGF-1 SDS value as independent covariate. Data in figure b are age-adjusted actual values. The primary efficacy analysis was based on the PP population and the supportive analysis on the ITT population. CI, confidence interval; IGF-1, insulin-like growth factor-1; ITT, intention to treat; LS, least squares; PP, per protocol; PR, prolonged release; SDS, standard deviation scores

LS mean (standard error) changes from baseline to the end-of-study/early withdrawal visit were comparable between lanreotide autogel and lanreotide 40 mg PR treatment groups. For the PP population, − 0.84 (0.15) (*n* = 54) compared with − 0.52 (0.17) (*n* = 44), respectively. For the ITT population, − 0.86 (0.13) (*n* = 61) compared with − 0.59 (0.14) (*n* = 60), respectively. The treatment difference (95% CI) between the lanreotide autogel and lanreotide 40 mg PR groups was − 0.32 (− 0.74, 0.11) in the PP population and − 0.27 (− 0.63, 0.09) in the ITT population. Non-inferiority of lanreotide autogel to lanreotide 40 mg PR was demonstrated in both the primary and supportive efficacy analyses as the upper limits of the 95% CIs in each case were below the pre-defined non-inferiority margin of 0.6 (Fig. 3a). A sensitivity analysis was performed using a mixed model repeated measures approach. For the PP population, the upper limit of the 95% CI for the treatment difference between lanreotide autogel and lanreotide 40 mg PR (without back transformation) was 0.16 (less than the non-inferiority margin of 0.6) and the upper limit of the 95% CI for the treatment ratio (with back transformation) was 1.17 (less than the non-inferiority margin of 1.822 [i.e. e(0.6)]). For the ITT population, the upper limit of the 95% CI for the treatment difference between lanreotide autogel and lanreotide 40 mg PR (without back transformation) was 0.14 (less than the non-inferiority margin of 0.6) and the upper limit of the 95% CI for the treatment ratio (with back transformation) was 1.15 (less than the non-inferiority margin of 1.822).

#### Actual age-adjusted IGF-1 SDS values

Values decreased for the actual age-adjusted IGF-1 SDS values throughout the study in each treatment group and for both the PP and ITT populations (Fig. 3b). Mean [95% CI] changes from baseline to the end-of-study/early withdrawal visit were similar for lanreotide autogel and lanreotide 40 mg PR. For the PP population, − 6.463 [− 8.261, − 4.665] (*n* = 57) and − 6.319 [− 8.219, − 4.418] (*n* = 48), respectively. For the ITT population the respective values were: − 6.453 [− 8.169, − 4.737] (*n* = 64) and − 7.003 [− 8.890, − 5.116] (*n* = 64). When the data were stratified by previous pituitary surgery, pituitary tumor type (i.e. microadenoma or macroadenoma), and sex, no notable differences were observed for the mean change-from-baseline to end-of-study/early withdrawal visit between the lanreotide autogel and lanreotide 40 mg PR groups (for both the PP and ITT populations).

#### Patient distribution by IGF-1 and GH levels at end-of-study/early withdrawal visit

**Fig. 4:**
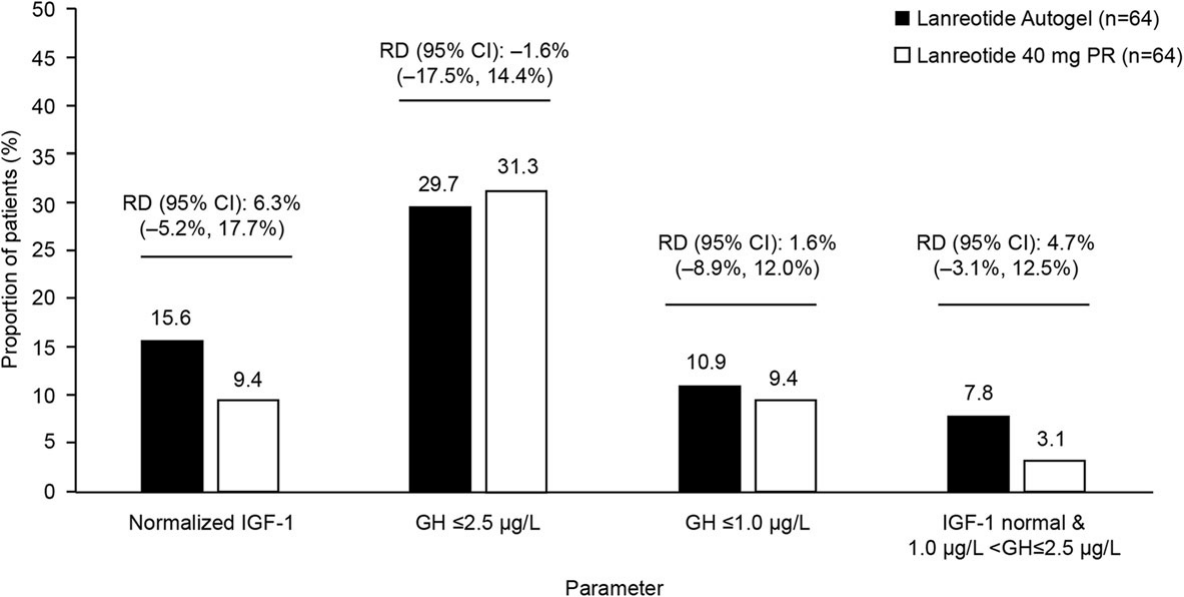
Patients achieving hormonal control at the end-of-study/early withdrawal visit. Data are from the intention-to-treat population. Decisions regarding the titration of treatment were made based on GH and IGF-1 levels measured at Week 13. CI, confidence interval; GH, growth hormone; IGF-1, insulin-like growth factor-1; PR, prolonged release; RD, risk difference

The proportion of patients (including risk difference [95% CI]) achieving hormonal control at the end-of study/early withdrawal in the lanreotide autogel and lanreotide 40 mg PR groups are shown in Fig. 4. Overall, 12.5% of patients had normal age-adjusted IGF-1 (15.6% in lanreotide autogel and 9.4% in lanreotide 40 mg PR), 30.5% of patients had GH levels ≤2.5 μg/L (29.7% in lanreotide autogel and 31.3% in lanreotide 40 mg PR), 10.2% of patients had GH levels ≤1.0 μg/L (10.9% in lanreotide autogel and 9.4% in lanreotide 40 mg PR), 5.5% of patients had normal IGF-1 and 1.0 μg/L < GH levels ≤2.5 μg/L (7.8% in lanreotide autogel and 3.1% in lanreotide 40 mg PR).

GH levels at end-of-study/early withdrawal visit decreased from baseline in both groups with mean [95% CI] decreases for lanreotide autogel and lanreotide 40 mg PR of − 9.548 μg/L [− 15.257, − 3.840] (*n* = 64) and − 13.182 μg/L [− 18.318, − 8.046] (*n* = 62), respectively. Mean [95% CI] difference between groups was 3.63 μg/L [− 3.98, 11.25].

#### Tumor volume reduction

**Fig. 5:**
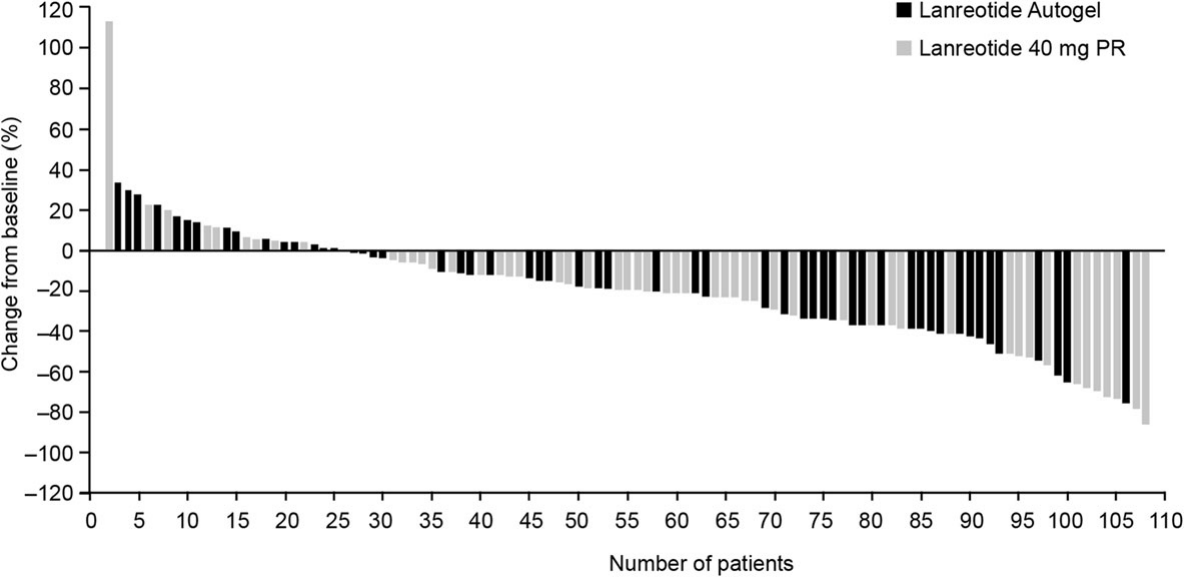
Reductions in tumor volume for individual patients. Data are change-from-baseline to end of study (or early withdrawal) in the solid component of the tumor for patients in the intention-to-treat population. One patient (in the lanreotide autogel 120 mg group) was classed as an outlier and his/her data are not shown in this figure

The proportions of patients achieving ≥20% reduction in the volume of the solid component of the tumor between baseline and the end-of-study/early withdrawal visit were similar in the two treatment arms: 45.5% (25/55) of patients in the lanreotide autogel group and 50.9% (27/53) of patients in the lanreotide 40 mg PR group. The risk difference between groups was − 5.5% [95% CI: –24.3, 13.3%]. Individual changes in tumor volume are shown in Fig. 5. Mean changes in tumor volume between baseline and the end-of-study/early withdrawal visit could not be compared across treatments as a result of the high variance in individual tumor volume. At the end-of-study/early withdrawal, median (range) changes were similar between lanreotide autogel, –17.9% (− 75.8, 243.9%; *n* = 54) and lanreotide 40 mg PR, − 20.1% (− 86.4, 113.0%; *n* = 53), an outlier in the lanreotide autogel 120 mg group had a positive percentage change in tumor volume of > 95,000% and was omitted from this analysis.

#### Acromegaly symptoms

Reductions in the proportions of patients with at least one acromegaly symptom were apparent during the study. At baseline, 95.3% (61/64) of patients in the lanreotide autogel group and 93.8% (60/64) of patients in the lanreotide 40 mg PR group had at least one acromegaly symptom. By week 13, this was reduced to 70.3% (45/64) and 64.4% (38/59; data missing for *n* = 5) of patients, respectively. At end-of-study/early withdrawal visit, 75.0% (48/64) and 61.3% (38/62; data missing for *n* = 2) of patients in the respective treatment groups had at least one acromegaly symptom.

### Safety

Overall, 123 patients (96.1%) in the safety population had TEAEs: 60 patients (93.8%) in the lanreotide autogel group and 63 patients (98.4%) in the lanreotide 40 mg PR group (Table 2). The TEAEs were predominantly of mild (41.4%) or moderate (46.9%) intensity. Two patients (3.1%) in the lanreotide autogel group and eight patients (12.5%) in the lanreotide 40 mg PR group had severe TEAEs. The most commonly reported TEAEs (> 10% incidence in either treatment group) were reported in a similar proportion of patients in the lanreotide autogel and the lanreotide 40 mg PR groups (Table 2). By contrast, headache, nausea, injection-site induration, injection-site pain and rectal tenesmus were reported by more patients in the lanreotide 40 mg PR group, and decreased appetite was reported in more patients in the lanreotide autogel group (between-group difference > 10%). Most TEAEs were considered related to treatment, with equal proportions of patients in each of the treatment groups reporting treatment-related TEAEs during the study (90.6% for both). The most common treatment-related TEAEs were of a similar nature to and were generally reported in a similar percentage of patients as the TEAEs described above.

**Table 2:** TEAEs

	**Lanreotide autogel** (***n*** **= 64**)^**a**^	**Lanreotide 40 mg PR** (***n*** **= 64**)
**Any TEAE**	60 (93.8)	63 (98.4)
Related to study treatment	58 (90.6)	58 (90.6)
Mild	25 (39.1)	28 (43.8)
Moderate	33 (51.6)	27 (42.2)
Severe	2 (3.1)	8 (12.5)
**Any serious TEAE**	1 (1.6)	2 (3.1)
Related to study treatment	0	1 (1.6)
**Any TEAE leading to withdrawal**	1 (1.6)	2 (3.1)
Related to study treatment	0	1 (1.6)
**TEAEs in > 10% of patients by preferred term**		
Diarrhea	51 (79.7)	46 (71.9)
Cholelithiasis	23 (35.9)	18 (28.1)
Abdominal distension	22 (34.4)	27 (42.2)
Upper respiratory tract infection	21 (32.8)	18 (28.1)
Abdominal pain	18 (28.1)	20 (31.3)
Upper abdominal pain	13 (20.3)	16 (25.0)
Hyperglycaemia	12 (18.8)	12 (18.8)
Headache^b^	10 (15.6)	17 (26.6)
Decreased appetite^c^	10 (15.6)	0
Sinus bradycardia	9 (14.1)	12 (18.8)
Weight decreased	8 (12.5)	4 (6.3)
Nausea^b^	7 (10.9)	16 (25.0)
Constipation	7 (10.9)	10 (15.6)
Nasopharyngitis	7 (10.9)	10 (15.6)
Blood glucose increased	7 (10.9)	6 (9.4)
Flatulence	7 (10.9)	3 (4.7)
Injection-site induration^b^	6 (9.4)	14 (21.9)
Dizziness	6 (9.4)	10 (15.6)
Alopecia	5 (7.8)	9 (14.1)
Thyroid nodule	4 (6.3)	7 (10.9)
Abnormal gastrointestinal sounds	3 (4.7)	9 (14.1)
Injection-site pain^b^	2 (3.1)	9 (14.1)
Rectal tenesmus^b^	1 (1.6)	9 (14.1)
Vomiting	1 (1.6)	7 (10.9)

Data are number (%) of patients from the safety population with AE classification from the Medical Dictionary for Regulatory Activities version 19.0. ^a^One patient randomly allocated to receive lanreotide 40 mg PR group incorrectly received lanreotide autogel at the first administration of study treatment. The patient experienced three AEs (gastrointestinal disorders of mild intensity and neither serious nor treatment-related); these AEs were considered to be prior AEs (i.e. not TEAEs) for lanreotide 40 mg PR and are not included in the above summary table

Between-group differences of > 10% are presented in the lanreotide 40 mg PR group (^b^) and the lanreotide autogel group (^c^)

*AE* adverse event, *PR* prolonged release, *TEAE* treatment-emergent AE

In total, nine patients had serious adverse events (SAEs) during the study (lanreotide autogel, *n* = 5; lanreotide 40 mg PR, *n* = 4); among them, three patients had serious TEAEs: one patient in the lanreotide autogel group as a result of a severe pituitary tumor (hypophysoma exacerbation), and two patients in the lanreotide 40 mg PR group as a result of cerebral infarction and gastritis. All three patients recovered from these serious TEAEs. Only the serious TEAE of gastritis was considered related to study treatment.

Three patients had TEAEs leading to study treatment withdrawal: 1 patient in the lanreotide autogel group (severe pituitary tumor) and 2 patients in the lanreotide 40 mg PR group (ECG QT interval prolonged and pregnancy). The patient with a severe pituitary tumor underwent surgery for removal of the tumor. The patient recovered from the serious TEAE and was discharged from the hospital. Study treatment was discontinued because of this serious TEAE; the investigator assessed the serious TEAE as not related to study treatment. One event reported in the lanreotide 40 mg PR group was considered related to study treatment (mild ECG QT interval prolonged; QT interval corrected by Fredericia was 464 ms at baseline; 484 ms at TEAE onset on Day 91; and 458 ms at the early withdrawal visit on Day 98). This patient recovered without medication intervention. No deaths were reported during the study.

#### Gallbladder echography

New incidences (i.e. was not present at baseline) of lithiasis or biliary sludge at end-of-study/early withdrawal visit were reported in 21 (32.8%) patients in the lanreotide autogel group and 16 (25.0%) patients in the lanreotide 40 mg PR group.

#### ECG and vital signs

The majority of patients had normal ECG evaluations at baseline (lanreotide autogel group [71.9%]; lanreotide 40 mg PR group [79.7%]). A slight decrease of mean heart rate from baseline was noted in both treatment groups at the Week 13 visit (lanreotide autogel group, − 8.5 beats/min; lanreotide 40 mg PR group, − 7.9 beats/min) and the end-of-study/early withdrawal visit (lanreotide autogel group, − 5.6 beats/min; lanreotide 40 mg PR group, − 3.6 beats/min). At the end-of-study/early withdrawal visit, four patients (6.3%) in the lanreotide autogel group and two patients (3.2%) in the lanreotide 40 mg PR group had a potentially clinically significant abnormality of low heart rate (≤50 bpm). A total of 21 patients (lanreotide autogel group, *n* = 9 [14.1%]; lanreotide 40 mg PR group, *n* = 12 [18.8]) overall reported TEAEs of sinus bradycardia during the study.

#### Glycaemia and HbA_1c_

In both the lanreotide autogel and lanreotide 40 mg PR groups, shifts from normal values at baseline to abnormally high value at Week 13 visit and end-of-study/early withdrawal visit were noted for fasting glucose and HbA_1c_ in some patients. At the end-of-study/early withdrawal visit, the proportion of patients with higher levels of fasting glucose were 28.1 and 20.3%, while the proportion with higher HbA_1c_ levels were 17.2 and 7.8%, in the lanreotide autogel group and the lanreotide 40 mg PR group, respectively. In the lanreotide autogel and lanreotide 40 mg PR groups, mean fasting glucose levels at the end-of-study/early withdrawal visit (6.18 mmol/L and 5.90 mmol/L, respectively) were slightly higher than they were at baseline (5.50 mmol/L and 5.63 mmol/L, respectively), whereas the mean HbA_1c_ was still within the normal range in both treatment groups.

#### Immunology

The presence of binding ADAs was infrequent; in the lanreotide autogel group, 3/63 (4.8%) patients were ADA positive at baseline and 2/63 (3.2%) patients seroconverted from negative at baseline to positive, both following treatment with lanreotide autogel 120 mg. For the lanreotide 40 mg PR group, 3/58 (5.2%) patients were ADA positive at baseline and 7/58 (12.1%) patients seroconverted from negative at baseline to positive following treatment every 7 days.

## Pharmacokinetics

At the end-of-study (i.e. at steady state), the mean (SD) lanreotide C_trough_ for patients receiving lanreotide autogel 120 mg after the fourth administration was 4.65 (1.86) ng/mL (*n* = 57), and the mean (SD) lanreotide C_trough_ for patients receiving lanreotide 40 mg PR every 7 days after the sixteenth administration was 2.62 (1.21) ng/mL (*n* = 50). Although the mean C_trough_ was statistically higher in patients receiving lanreotide autogel 120 mg than in those receiving lanreotide 40 mg PR every 7 days, variability was high and a significant overlap was observed between the groups. As there was no observable impact on change in IGF-1 or GH control, the difference in C_trough_ at steady state between the two formulations is not considered clinically significant.

After the fourth titrated dose of lanreotide autogel 120 mg, the mean (SD) C_max_ was 13.6 (6.51) ng/mL in the PK subgroup (*n* = 15), which was reached at a median time of 8 h (t_max_). In this subgroup, mean C_trough_ (serum concentration at 28 days [672 h] post-dose) was 4.40 (2.60) ng/mL. The mean AUC_tau_ was 3966 ng*h/mL and the median apparent elimination t_½_ was 36.5 days.

Accumulation of lanreotide was evaluated by comparing C_trough_, C_max_ and AUC_tau_ obtained after the fourth titrated dose of 120 mg lanreotide (normalized to a dose of 90 mg) and those obtained after the first dose of 90 mg in order to calculate the R_trough_, R_max_, and R_AUC_. Modest drug accumulation was apparent after repeated dosing based on mean R_trough_, R_max_, and R_AUC_ of 2.62, 1.40, and 2.33, respectively.

## Discussion

Lanreotide autogel is a SSA approved for the treatment of acromegaly in 73 countries, including 27 European countries, Japan, and the US. However, as of 2019 it is not yet approved for use in Chinese patients with acromegaly. In this phase 3, prospective, randomized, stratified, parallel-group, non-inferiority study, Chinese patients with active acromegaly were randomized to either lanreotide autogel 60, 90, or 120 mg or lanreotide 40 mg PR. Over the study period, lanreotide autogel demonstrated comparable levels of hormonal control, tumor volume reduction and management of acromegaly symptoms to lanreotide 40 mg PR. In addition, the safety data were consistent with the known profile of lanreotide autogel.

Lanreotide autogel was non-inferior to lanreotide 40 mg PR in Chinese patients with acromegaly in terms of IGF-1 SDS values. The proportions of patients achieving hormonal control were similar for lanreotide autogel and lanreotide 40 mg PR. Almost one third of patients had achieved GH levels ≤2.5 μg/L and more than 10% had achieved normalized IGF-1 levels by end-of-study/early withdrawal visit in both groups. The proportions of patients reaching both a normal IGF-1 and a 1.0 μg/L < GH ≤2.5 μg/L, however, were less than 10% in each group. Previous European studies have reported hormonal control (GH levels < 2.5 ng/mL and age−/sex-normalized IGF-I) in 33 and 39% of patients receiving lanreotide 30 mg microparticles (after the fifth dose) and lanreotide autogel (after the third dose), respectively [12]. The latter study was followed by a 1-year extension study, where 32% of patients in the lanreotide 30 mg microparticles group and 43% of patients in the lanreotide autogel group achieved hormonal control (GH ≤2.5 μg/L and normalized IGF-I) after 1 year of titrated-dose of lanreotide autogel [13]. After 3 years of titrated treatment with lanreotide autogel, hormonal control (GH ≤2.5 μg/L and age- and sex-normalized IGF-1) was achieved in 46% of patients [15]. The lower proportion of patients achieving this level of hormonal control in the present study compared with those cited could be as a result of a number of factors including the use of different assays for IGF-1, prior radiotherapy, differences in the inclusion/exclusion criteria, and the proportion of patients who had previously received acromegaly treatment or were receiving acromegaly treatment at study inclusion. Future studies could involve stratified analyses of these factors to elucidate the impact they may have on hormonal control. Another factor that may impact hormonal control is patient resistance to first-generation SSAs. Colao et al. (2011) suggested that of those patients who did not achieve a biochemical or a tumor response during SSA treatment, < 10% could be considered resistant to treatment; however, this was not investigated in the LANTERN study [20].

In this study, 45.5% (lanreotide autogel) and 50.9% (lanreotide 40 mg PR) of patients achieved a ≥ 20% reduction in solid tumor component volume between baseline and the end-of-study/early withdrawal visit. Similar proportions have been reported in other studies. In the PRIMARYS study, the proportion of treatment-naïve patients with a ≥ 20% tumor volume reduction was 54.1% at Week 12 and 56.3% at Week 24 [17].

We observed reductions in the proportions of patients with at least one acromegaly symptom in both treatment groups by Week 13 of the LANTERN study. Other studies have also observed an improved control or reduction in acromegaly symptoms with lanreotide autogel treatment that was comparable or greater than with lanreotide 30 mg [12, 13, 15, 17].

In the present study, the most common TEAEs in the lanreotide autogel group included diarrhea, abdominal distension, abdominal pain and cholelithiasis. These were also the most commonly reported TEAEs in the lanreotide 40 mg PR group. No new safety signals were observed in the present study. These safety findings are also consistent with previous European studies of lanreotide autogel in post-surgical patients [12, 13] and treatment-naïve patients [17] with acromegaly. In addition, these AEs are all ‘commonly’ or ‘very commonly’ reported with lanreotide autogel according to the European summary of product characteristics (SmPC) [9] and US prescribing information [10]. Gastrointestinal AEs are known side effects of SSA therapy, and have also been reported with octreotide [21] and pasireotide treatment [22].

Steady-state PK was achieved after the administration of four lanreotide autogel doses. In the present study, after the fourth administration, patients receiving lanreotide autogel 120 mg had a mean C_trough_ of 4.65 ng/mL (*n* = 57). Similarly, Bronstein and colleagues reported a steady state C_min_ (equivalent to C_trough_) of 3.76 ng/mL [19]. In this study, modest drug accumulation was apparent after repeated dosing, which is in line with the available data for Caucasian patients with acromegaly, where a mean accumulation ratio index of 2.7 (which is in line with range of values for the half-life of lanreotide) was reported [10]. According to PK modeling, these serum lanreotide concentrations are adequate to maintain GH suppression [23].

In this study, doses of lanreotide autogel were administered in accordance with the US prescribing information [10], with an initial starting dose of 90 mg at 4-week intervals for 3 months, and the European SmPC, which recommend a starting dose of 60 to 120 mg every 28 days [9]. Thereafter, patients were individually titrated to determine the clinically effective dose based upon reduction in biochemical markers (GH and IGF-1) and symptoms, in line with the European SmPC and US prescribing information. Following the 3-month fixed-dose period, the majority of patients were up-titrated to 120 mg of lanreotide autogel. Dose titration has previously been demonstrated as an effective mechanism for improved hormonal and symptom control in acromegaly [13]. Thus, dosing regimens tailored specifically for the Chinese population are not required. Both the European SmPC and US prescribing information recommend that patients with acromegaly who are well-controlled can be considered for an extended dosing interval of lanreotide autogel 120 mg every 6–8 weeks.

One of the limitations of the present study was the open-label design, which meant patients and physicians were aware of the allocated treatment and thus had the potential to introduce reporting bias. The population also comprised patients with a mixed treatment history, which, while a limitation of the study, also demonstrates the real-world patient experience. These limitations notwithstanding, the LANTERN study benefitted from a randomized design, centralized blinded review of MRI scans, and an objective primary endpoint for assessment. A sensitivity analysis proved the robustness of the primary analysis.

## Conclusions

Lanreotide autogel (monthly deep subcutaneous injection) was non-inferior to lanreotide 40 mg PR (intramuscular injection every 7, 10, or 14 days) in terms of efficacy for the treatment of active acromegaly in Chinese patients. No meaningful differences between treatments were apparent for hormonal control, tumor volume, and acromegaly symptoms. The PK profiles of lanreotide autogel in Chinese patients with acromegaly demonstrated sufficient exposure to adequately maintain IGF-1 and GH suppression. Lanreotide autogel is therefore suitable for dosing at 4-week intervals in Chinese patients with acromegaly. Overall, the safety profile of lanreotide autogel was similar to that for lanreotide 40 mg PR in the Chinese patients studied. It was also consistent with previous reports in other populations, and the US and European prescribing guidelines.

## Supplementary information

**Additional file 1.** Protocol amendments.

## Data Availability

Where patient data can be anonymized, Ipsen will share all individual participant data that underlie the results reported in this article with qualified researchers who provide a valid research question. Study documents, such as the study protocol and clinical study report, are not always available. Proposals should be submitted to DataSharing@Ipsen.com and will be assessed by a scientific review board. Data are available beginning 6 months and ending 5 years after publication; after this time, only raw data may be available.

## Abbreviations

ADA: Anti-drug antibodies
AUC_tau_: Area under the concentration–time curve over a 4-week dosing interval
CI: Confidence interval
C_max_: Maximum plasma concentration
C_trough_: Trough lanreotide concentrations
ECG: Electrocardiogram
ECL: Electrochemiluminescence
GCP: Good Clinical Practice
GH: Growth hormone
GLM: Generalized linear model
HbA_1c_: Glycated hemoglobin
IGF-1: Insulin-like growth factor
ITT: Intention-to-treat
LAR: Long-acting release
LLN: Lower limit of normal
LS: Least squares
MRI: Magnetic resonance imaging
PK: Pharmacokinetic
PP: Per protocol
PR: prolonged release
R_AUC_: Accumulation ratio for AUC_tau_
R_trough_: Accumulation ratio for C_trough_
SAE: Serious adverse event
SD: Standard deviation
SDS: Standard deviation scores
SSA: Somatostatin analog
TEAE: Treatment-emergent adverse event
t_max_: Time to maximum plasma concentration
TV: Tumor volume
ULN: Upper limit of normal
