# Supplementary material for: Efficacy and safety of lanreotide autogel compared with lanreotide 40 mg prolonged release in Chinese patients with active acromegaly: results from a phase 3, prospective, randomized, and open-label study (LANTERN)
Source: BMC Endocr Disord. 2020 May 4;20:57. doi: 10.1186/s12902-020-0524-7 (PMC7199333; doi:10.1186/s12902-020-0524-7)
Supplement: Supplementary file 1 — Additional file 1. Protocol amendments. [file 12902_2020_524_MOESM1_ESM.docx]

**Efficacy and safety of lanreotide autogel compared with lanreotide 40 mg prolonged release in Chinese patients with active acromegaly: Results from a phase 3, prospective, randomized, and open-label study (LANTERN)**

**Additional file 1**

*Protocol amendments*

The protocol was finalized on 26 March 2014. Key amendments to the protocol are as follows:

Addition of exclusion # 13, i.e. replacement of a single random growth hormone (GH) sample by a GH cycle (sample taken at 0, 30, 60, 90 and 120 minutes);

Replacement of “age- and sex-adjusted insulin-like growth factor-1 (IGF-1)” with “age-adjusted IGF-1” to align the protocol with current knowledge on this particular topic within this disease area.
